# Crystal structures of glutathione- and inhibitor-bound human GGT1: critical interactions within the cysteinylglycine binding site

**DOI:** 10.1074/jbc.RA120.016265

**Published:** 2020-11-22

**Authors:** Simon S. Terzyan, Luong T. Nguyen, Anthony W.G. Burgett, Annie Heroux, Clyde A. Smith, Youngjae You, Marie H. Hanigan

**Affiliations:** 1 Laboratory of Biomolecular Structure and Function, Department of Biochemistry and Molecular Biology, University of Oklahoma Health Sciences Center, Oklahoma City, Oklahoma, USA; 2 Department of Pharmaceutical Sciences, The State University of New York at Buffalo, Buffalo, New York, USA; 3 Department of Pharmaceutical Sciences, College of Pharmacy, University of Oklahoma Health Sciences Center, Oklahoma City, Oklahoma, USA; 4 Energy Sciences Directorate/Photon Science Division, Brookhaven National Laboratory, Upton, New York, USA; 5 Stanford Synchrotron Radiation Lightsource, Stanford University, Menlo Park, California, USA; 6 Department of Cell Biology, University of Oklahoma Health Sciences Center, Oklahoma City, Oklahoma, USA

**Keywords:** crystal structure, enzyme, enzyme inhibitor, crystallography, structural biology, γ-glutamyl transpeptidase, γ-glutamyl transferase, glutathione, ACPB, 2-amino-4-(((1-((carboxymethyl)amino)-1-oxobutan-2-yl)oxy)(phenoxy)phosphoryl)butanoic acid, GGT1, γ-glutamyl transpeptidase aka γ-glutamyl transferase, GSH, glutathione, GSSG, oxidized glutathione, ɣ-Glu AMC, L-γ-glutamyl 7-amido-4-methylcoumarin, GpNA, γ-glutamyl p-nitroanilide, hGGT1, human GGT1, LTC4, leukotriene C4, PDB, Protein Data Bank, RMS, root mean square

## Abstract

Overexpression of γ-glutamyl transpeptidase (GGT1) has been implicated in an array of human diseases including asthma, reperfusion injury, and cancer. Inhibitors are needed for therapy, but development of potent, specific inhibitors of GGT1 has been hampered by a lack of structural information regarding substrate binding and cleavage. To enhance our understanding of the molecular mechanism of substrate cleavage, we have solved the crystal structures of human GGT1 (hGGT1) with glutathione (a substrate) and a phosphate-glutathione analog (an irreversible inhibitor) bound in the active site. These are the first structures of any eukaryotic GGT with the cysteinylglycine region of the substrate-binding site occupied. These structures and the structure of apo-hGGT reveal movement of amino acid residues within the active site as the substrate binds. Asn-401 and Thr-381 each form hydrogen bonds with two atoms of GSH spanning the γ-glutamyl bond. Three different atoms of hGGT1 interact with the carboxyl oxygen of the cysteine of GSH. Interactions between the enzyme and substrate change as the substrate moves deeper into the active site cleft. The substrate reorients and a new hydrogen bond is formed between the substrate and the oxyanion hole. Thr-381 is locked into a single conformation as an acyl bond forms between the substrate and the enzyme. These data provide insight on a molecular level into the substrate specificity of hGGT1 and provide an explanation for seemingly disparate observations regarding the enzymatic activity of hGGT1 mutants. This knowledge will aid in the design of clinically useful hGGT1 inhibitors.

γ-glutamyl transpeptidase (GGT1, aka γ-glutamyl transferase) is a cell surface enzyme that cleaves extracellular glutathione (GSH), GSH *S*-conjugates, and other γ-glutamyl compounds (1, 2). Overexpression of GGT1 contributes to the severity of a variety of pathological conditions including asthma, reperfusion injury, and cardiovascular disease (3, 4, 5, 6). It stimulates tumor growth and activates compounds including the chemotherapy drug cisplatin to nephrotoxins (7, 8, 9, 10, 11). Inhibiting the enzyme would be therapeutic in the treatment of multiple diseases, and development of novel GGT1 inhibitors has been the focus of intense research (12, 13, 14). Most GGT1 inhibitors are glutamate analogs and bind the γ-glutamyl binding region within the active site. These inhibitors also block essential glutamine and glutamate-metabolizing enzymes making them too toxic for clinical use (15, 16). In order to develop more specific and less toxic inhibitors, there have been efforts to utilize interactions in the region of the active site of GGT that binds the cysteinylglycine moiety of GSH (13, 17). However, there is a lack of structural information regarding the interactions between substrates/inhibitors and the enzyme within the cysteinylglycine binding region of human GGT1 (hGGT1). The structures solved in this study include substrate–hGGT1 interactions within this region of the active site. Analysis of these structures in combination with our previously reported structures identifies, for the first time, movement within the active site during substrate/inhibitor binding.

We previously reported the crystal structure of hGGT1 (Fig. 1) (18). hGGT1 is translated as a single polypeptide chain, which folds and autocleaves into two subunits with Thr-381 as the N-terminus of the small subunit (19, 20). The side-chain oxygen of Thr-381 is the nucleophile in hGGT1-catalyzed reactions (Fig. 2). Amino acids from both the large subunit (amino acids 1–380) and small subunit (amino acids 381–569) are present within the active site of the enzyme. We have solved the structures of the apo-enzyme (4Z9O), hGGT1 with glutamate bound in the active site (4ZCG), and hGGT1 inhibited by a series of glutamate analogs, including serine-borate (4Z6C), GGsTop (4ZBK), and diazonorleucine (5V4Q) (18, 21, 22). Our structures show that γ-glutamyl compounds are bound within the active site by a network of salt bridges and hydrogen bonds between their α-carboxy and α-amine groups and the enzyme. The mechanism by which hGGT1 cleaves γ-glutamyl bonds is shown in Figure 2. Briefly, an oxyanion hole (Gly-473 and Gly-474) facilitates the attack of the side-chain oxygen of Thr-381 on C5 (carboxyl carbon) of the substrate’s γ-glutamyl group. The formation of this C–O bond results in a tetrahedral intermediate. Upon formation of this intermediate, the γ-glutamyl bond of the substrate (one of the four bonds of the tetrahedral intermediate) is hydrolyzed, and all of the substrate, except the γ-glutamyl group, is released from the enzyme (first product of the reaction). Cleavage of the covalent acyl bond between Thr-381 of the enzyme and the C5 of the γ-glutamyl group is the rate-limiting step in the catalytic reaction. Hydrolysis of this acyl bond yields glutamate and the free enzyme. Alternatively, in the presence of high, nonphysiological concentrations of acceptor dipeptides, the acyl bond can be resolved by the transfer of the γ-glutamyl group to an acceptor amine, resulting in a transpeptidation reaction (Fig. 2). The transpeptidation reaction yields a new γ-glutamyl compound and the free enzyme. Kinetic studies indicate that the substrates and inhibitors that contain a cysteinylglycine moiety have enhanced affinity for the human enzyme (13, 17). Our novel structures show the molecular interactions between the enzyme and the cysteinylglycine moiety of both GSH and 2-amino-4-(((1-((carboxymethyl)amino)-1-oxobutan-2-yl)oxy)(phenoxy)phosphoryl)butanoic acid (ACPB), a GSH analog.

**Figure 1:**
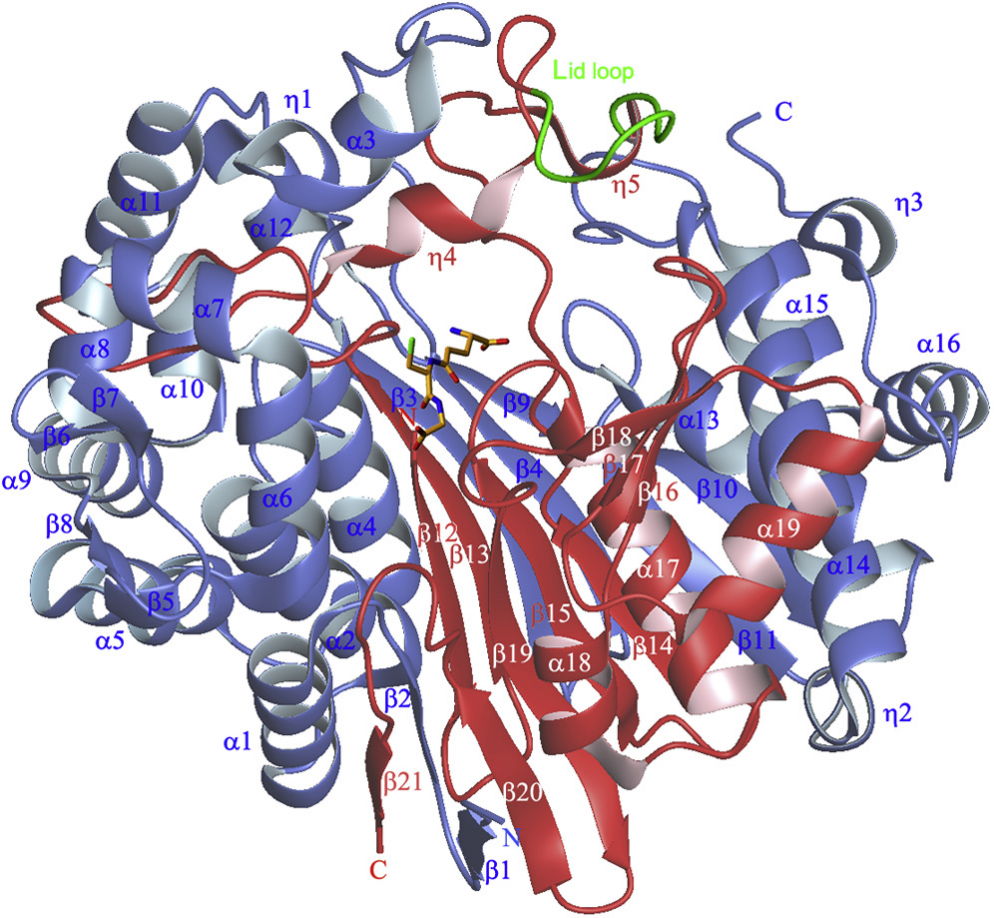
**A ribbon presentation of apo-hGGT1 (4Z9O) with GSH (a substrate) inserted to show the location of the active site.** The large subunit is colored *blue*, the small subunit is colored *red*. The *green* ribbon highlights the lid loop region of the small subunit. GSH carbon atoms are colored *orange*, oxygens are *red*, nitrogens are *blue*, and sulfur is *green*. (See Table S1 for sequences of each structural element.)

**Figure 2:**
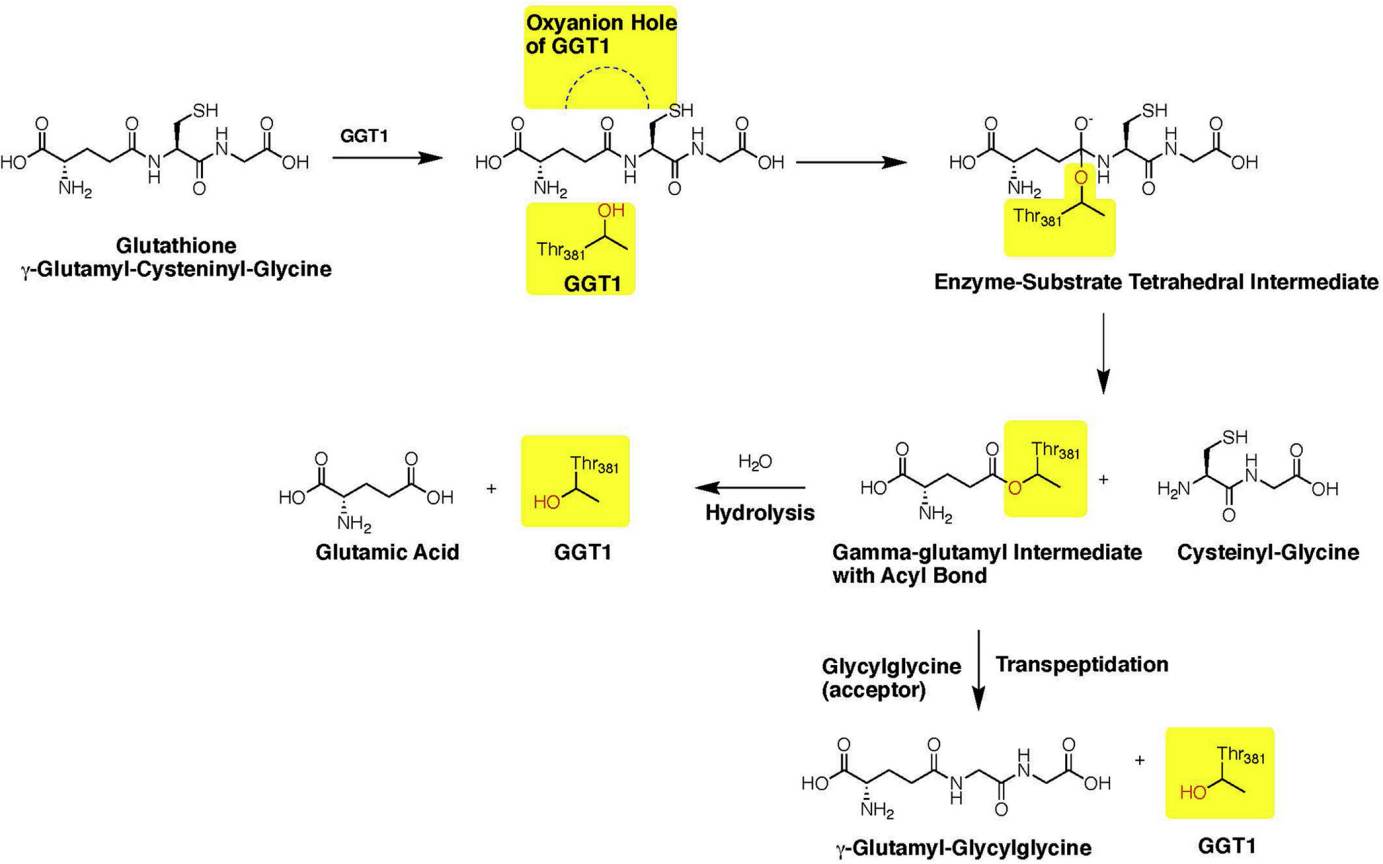
**Hydrolysis and transpeptidation reactions catalyzed by hGGT1.***Yellow highlight* denotes atoms of the enzyme. Nonhighlighted atoms are the substrate, GSH.

In this study, we report the first crystal structure of hGGT1 with GSH in the active site. GSH is the most abundant physiological substrate of the enzyme. In addition, we solved the structure of hGGT1 inhibited with the phospho-glutathione analog ACPB, a GGT1 inhibitor first reported by Han and colleagues (Fig. 3) (17). These are the first structures of any eukaryotic GGT with the cysteinylglycine region of the substrate-binding site occupied. These data reveal previously unknown interactions between the GSH and the enzyme. Comparison of our apo-hGGT1 structure (4Z9O) with the GSH-bound hGGT1 and ACPB-bound hGGT1 structures identified movement both within structural regions of the active site and amino acid side chains during substrate/inhibitor binding. These data provide new information about the molecular mechanism of substrate and inhibitor binding in the active site of hGGT1. The data also provide insight into changes in the enzymatic activity observed in hGGT1 mutants (17, 23, 24, 25, 26).

**Figure 3:**
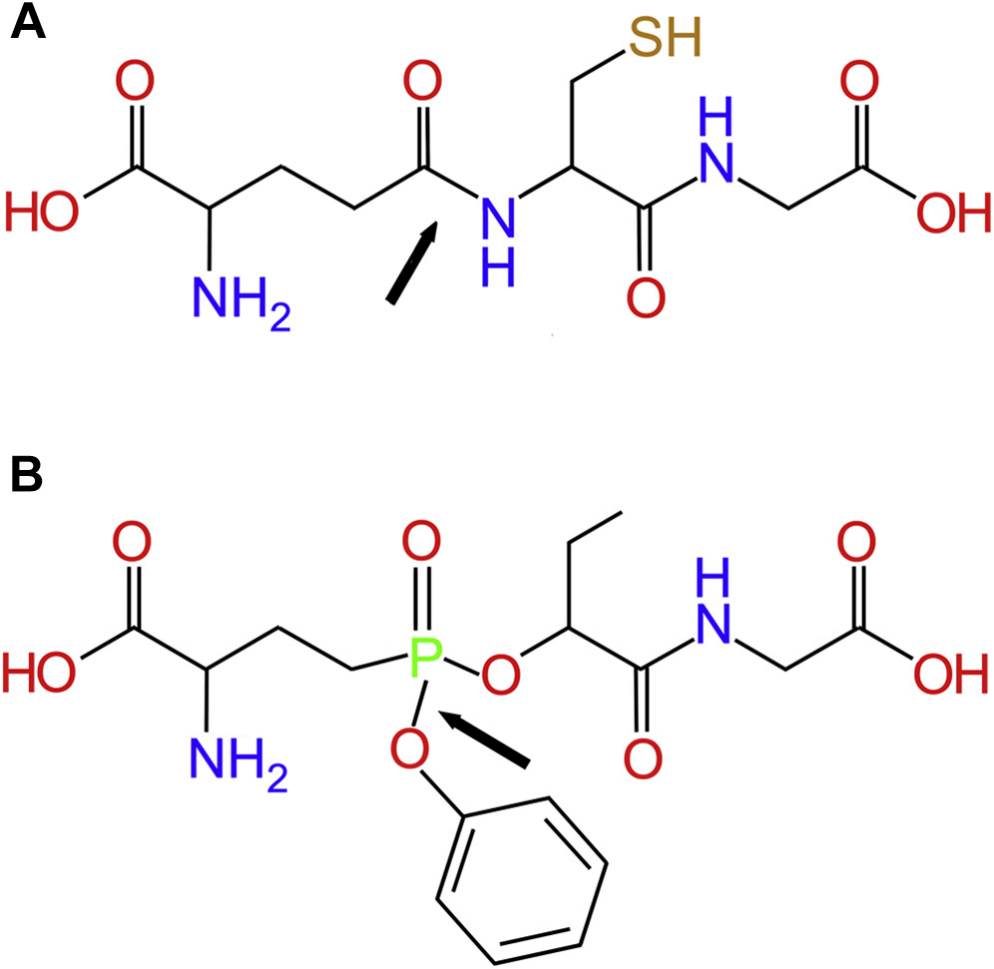
**GSH and the GGT1 inhibitor ACPB**. *A*, structure of GSH. *Arrow* indicates the γ-glutamyl bond of GSH cleaved by GGT1. *B*, structure of ACPB. *Arrow* indicates the bond that is cleaved as ACPB binds to and inactivates the enzyme. The phenoxy group is the leaving group and is not seen in the structure of ACPB-bound hGGT1 (Fig. 6).

## Results and discussion

### Structure of GSH-bound hGGT1

**Figure 4:**
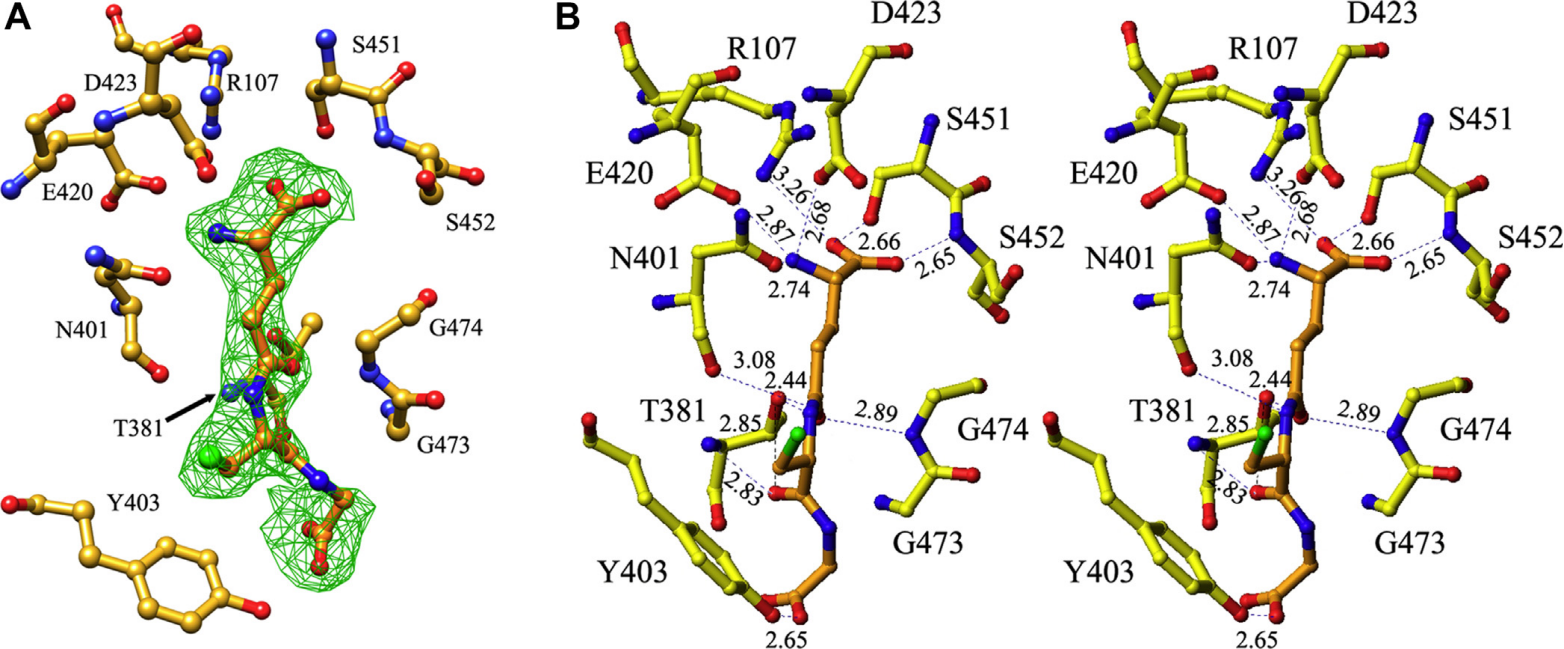
**GSH in the active site of hGGT1 (6XPC).***A*, the density from the *F*_*o*_*-F*_*c*_ map fitted with the GSH molecule. *B*, relaxed stereo presentation of the final model of GSH-bound hGGT1. Enzyme carbon atoms are colored *yellow*; GSH carbon atoms are colored *orange*, oxygens are *red*, nitrogens are *blue*, and sulfur is *green*. Resolution is 2.26 Å.

**Figure 5:**
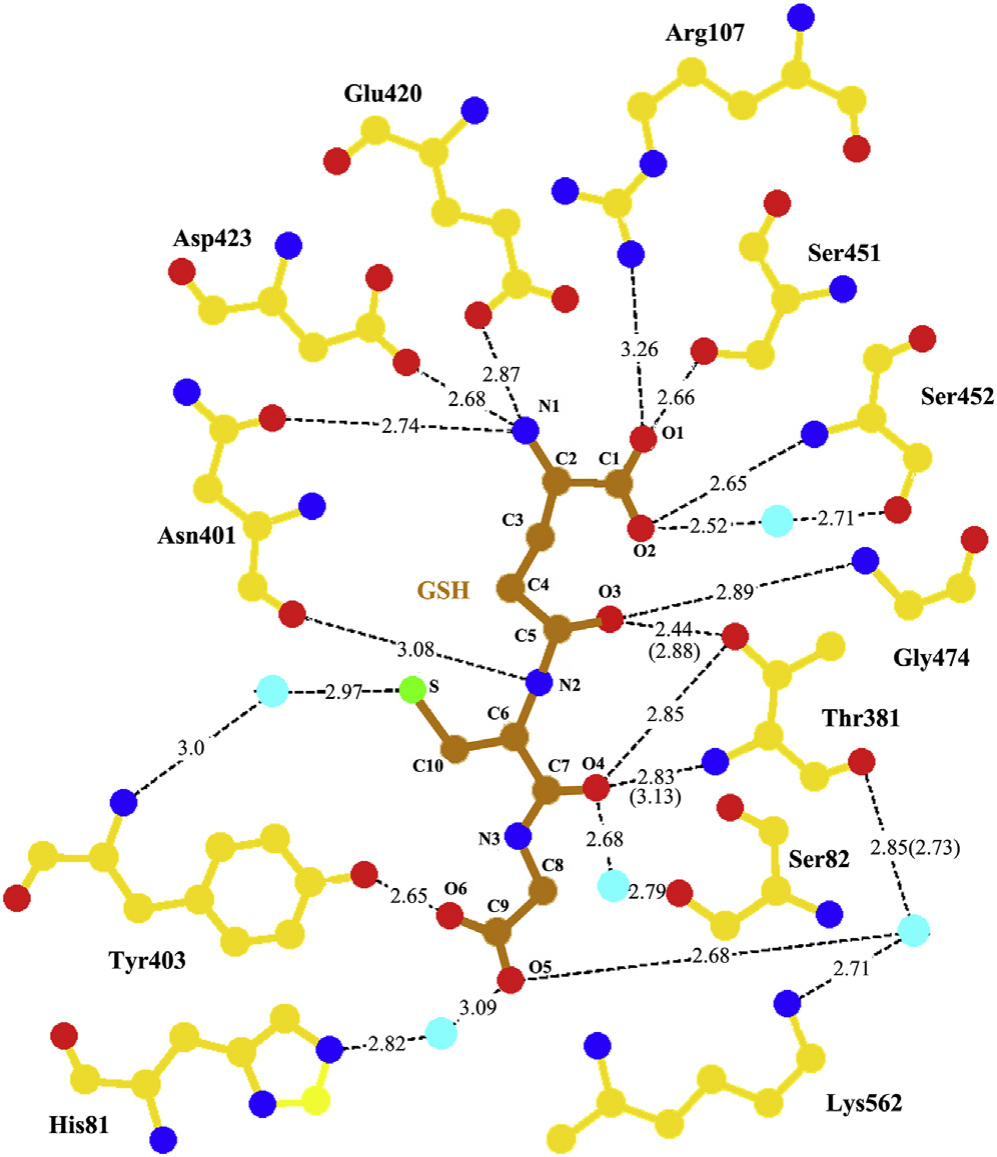
**LIGPLOT diagram of the interactions between hGGT1 and GSH.** Bond lengths to Thr-381 are conformation A (conformation B). Enzyme carbon atoms are colored *yellow*; GSH carbon atoms are colored *orange*, oxygens are *red*, nitrogens are *blue,* sulfur is *green,* and water molecules are *cyan*.

Crystals of apo-hGGT1 were soaked for 2 h in mother liquor containing 200 mM oxidized GSH (GSSG). The resolution of the data set was truncated at 2.26 Å based on I/σ, R_merge_ and completeness in last resolution shell (Table 1). After the initial ten cycles of rigid body and ten cycles of restrained refinement of the coordinates of apo-hGGT1 (4Z9O), (containing only coordinates of protein and carbohydrates with no alternative conformations), *F*_*o*_*-F*_*c*_ and 2*F*_*o*_*-F*_*c*_ maps were calculated. They showed clear electron density for a complete GSH molecule in the active site (Fig. 4 and Fig. 5). An intact molecule of GSH was fitted into the difference density and subsequently refined together with the hGGT1 molecule. In the final structure (with R_work_ = 16.49 and R_free_ = 20.89), no acyl bond was detected between Thr-381 and the GSH molecule. The catalytic nucleophile Thr-381 had two conformations with chi1 angles of 79.96° (conformation A) and –171.5° (conformation B), similar to those observed previously in apo-hGGT1 (4Z9O) (21). Conformation A is the same as the conformation of the Thr-381 in the structures with glutamate-based inhibitors in which an acyl bond was detected (21, 22). GSSG, the oxidized form of GSH, was added to the crystals, but only GSH was observed in the structure. There was no density to indicate the presence of a disulfide bond. No covalent bonds, but numerous hydrogen bonds and salt bridges, formed between the GSH molecule and the enzyme (Fig. 4*B* and Fig. 5).

**Table 1:** Diffraction data and refinement statistics—values in parenthesis refer to the highest resolution shell

Name	GSH-bound hGGT1	ACPB-bound hGGT1
Data Collection		
PDB code	6XPC	6XPB
Space Group	C*222*_1_	C*222*_1_
Unit cell (Å)	105.4 124.7 105.0	106.4 124.2 104.2
Resolution (Å)	20–2.26 (2.39–2.25)	64–1.74 (1.77–1.74)
No. of reflections	32,583	69,800 (3284)
Data cutoff	-3σ	-3σ
Completeness (%)	98.8 (93.8)	98.3 (93.6)
Redundancy	6.47 (6.08)	6.9 (5.0)
<I>/<*σ*>	15.01 (2.52)	15.7 (1.8)
*R*_merge_(%)^a^	8.1 (62%)	12.1 (64.2)
CC_1/2_	99.9 (87.8)	(70.0)
*B* factor from Wilson plot (Å^2^)	47.9	16.6
Refinement		
Resolution (Å)	20–2.26 (2.32–2.26)	63.9–1.74 (1.78–1.74)
No. of reflections		
Work set	30,953 (2197)	66,262 (3533)
Free set	1630 (116)	4604 (243)
No. of atoms (total)	4492	5040
*R*_work_ (%)	16.49 (29.6)	15.32 (30.4)
*R*_free_ (%)	20.89 (31.7)	18.89 (31.4)
*R*_overall_ (%)	16.7	15.5
Figure of merit	82.26	82.44
Correlation coefficient	97.0	97.1
Mean *B* (Å^2^)		
All	42.8	24.4
Protein		
A chain	42.7	22.3
B chain	40.1	18.8
Water	48.3	42.8
ACPB	59.0	22.0
Cofactors (Cl, Na)	30.8	31.7
Carbohydrate	73.1	43.5
Estim. coord. error based on likel. (Å)	0.149	0.063
Estim *B* value error (Å^2^)	6.38	2.0
*RMS* from ideal values		
Bonds	0.007	0.013
Angles	1.15	1.61

Bwilson, Bfactor determined from Wilson plot.

a *R*_merge_ = *(Σ*_*H*_*Σ*_*j*_*|I*_*hj*_*− <I*_*H*_*>|)/(Σ*_*H*_*Σ*_*j*_*<I*_*H*_*>)* where *I*_*Hj*_ is *j*th observation of reflection *H*.

The enzyme–substrate interactions within the glutamate binding region of the active site replicated those we have observed previously in our structures of glutamate or glutamate analogs bound to hGGT1 (18, 21, 22). These bonds are shown in Figures 4 and 5, and Movie S1. One of the carboxy oxygens (O1) formed a hydrogen bond with the side-chain oxygen of Ser-451 and salt bridge with the side-chain nitrogen of Arg-107. The other carboxy oxygen (O2) formed a hydrogen bond with the main-chain nitrogen of Ser-452 and interacted through a water molecule with the side-chain oxygen of Ser-452. The α-nitrogen (N1) of the glutamate formed a hydrogen bond with the side-chain oxygen of Asn-401 and two salt bridges with the side-chain oxygens of Asp-423 and Glu-420.

Of particular interest are the initial interactions between GSH and the enzyme in proximity to the γ-glutamyl bond of GSH (see Fig. 3 [arrow] and Fig. 5). The catalytic activity of the enzyme is the cleavage of this bond (Fig. 2). The carbonyl oxygen (O3) on the C5 carbon of the substrate interacts with the backbone nitrogen of Gly-474, one of the two Gly residues of the oxyanion hole (Fig. 4*B* and Fig. 5). Seen for the first time are two residues, Asn-401 and Thr-381, interacting with atoms of GSH that span the γ-glutamyl bond (Movie S1). In Movie S1, for clarity, Thr-381 is shown only in conformation A. As noted above, Asn-401 interacts with the α-nitrogen (N1) of the glutamate of GSH. In addition, the main chain oxygen of Asn-401 forms a hydrogen bond with the nitrogen of the γ-glutamyl bond of GSH (N2). Thr-381 interacts with the O3 and O4 of GSH, oxygens on opposite sides of the γ-glutamyl bond (Fig. 5). These interactions, in concert within the cysteinylglycine binding region (described below), may begin to orient the substrate and aid in drawing it further into the active site.

The GSH-bound hGGT1 complex revealed, for the first time, interactions between the enzyme and substrate within the cysteinylglycine binding region of the active site. Hydrogen bonds were observed between the enzyme and five atoms of the cysteinylglycine of GSH, including the α-nitrogen, sulfur and carboxyl oxygen of the cysteine, as well as both carboxyl oxygens of glycine (Fig. 4 and Fig. 5). The carboxyl oxygen (O4) of the cysteine of GSH was within hydrogen bonding distance of three atoms of the enzyme: the side-chain oxygen of Thr-381 in conformation B (2.85 Å); the α-nitrogen of Thr-381 (2.83 Å for conformation A and 3.13 Å for conformation B); and through a water molecule the carbonyl oxygen of cysteine interacted with side-chain OG atom of Ser-82. This extensive bonding suggests that this oxygen contributes to the affinity of the substrate for the enzyme and alignment of the substrate in the active site. The sulfur of the cysteine was bound through a water molecule (2.97 Å) to the α-nitrogen of Tyr-403 (3.0 Å). One of the carboxy oxygens (O6) of the glycine portion of GSH was within hydrogen bonding distance of the side-chain hydroxyl of Tyr-403 (2.65 Å). The interactions of the second carboxy oxygen of glycine (O7) with the protein were mediated by two water molecules. One of the water molecules (distance 2.68 Å) interacted with the NZ atom of Lys-562 (2.71 Å) and the carbonyl oxygen of Thr-381 (2.73 Å for conformation A and 2.85 Å for conformation B), while it interacted through a second water molecule (3.09 Å) with the NE2 atom of His-81 (2.82 Å).

### Movement within the active site of hGGT1 as GSH binds

LSQ superposition of the structures of the GSH-bound hGGT1 complex (6XPC) and the apo-hGGT (4Z9O) revealed minor shifts in the polypeptide backbone of the enzyme as GSH bound in the apo-hGGT1 crystals (Movie S2). In the apo-hGGT1 structure reported previously, we found that the N-terminus (amino acids 28–32) and the C-terminus (amino acids 375–380) of the large subunit were disordered. Therefore, we superimposed the CA atoms of 342 residues of the large subunit (amino acids 34–375) of the apo- and GSH-bound hGGT1 structures. The *rms* deviation was 0.22 Å with largest shift of 1.51 Å located at the N terminus of large subunit, which is adjacent to the region that was previously found to be disordered. When three additional N- and C- terminal residues (34, 35, and 375) were excluded from superposition, the *rms* deviation for the CA atoms of the large subunit was 0.19 Å, with maximum deviation of 0.6 Å. We superimposed the CA atoms of all 189 residues of the small subunit (amino acids 381–569). The *rms* deviation was 0.29 Å with maximum deviation of 1.68 Å for the CA atom of Thr-475. This threonine residue is part of the oxyanion-hole forming loop that participates in the catalytic activity of the enzyme, but Thr-475 had no direct contact with the substrate. In comparison with the apo-hGGT1 structure, Thr-475 in the GSH-bound hGGT1 structure had moved away from the bound substrate molecule. The CA atoms of Gly-473 and Gly-474 were shifted 1.37 Å and 1.49 Å, respectively (Movie S2). The displacement of the oxyanion loop (compared with our apo-hGGT1 structure (4Z9O) was the dominant shift observed upon GSH entering the active site and demonstrates the flexibility of this loop even in crystals of hGGT1.

This structure suggests that the GSH molecule is aligned within the active site, with the glutamate portion bound; however, the crystals may have been too rigid for the conformational shifts required for tight binding of GSH within the active site. The crystals of GSH-bound hGGT1 were prepared by soaking the crystals of the apo-hGGT1 in GSH. We propose that our structure is an intermediate structure in the binding of GSH in the active site of hGGT1. Data from the ACPB-bound hGGT1 structure below provide additional support for this hypothesis.

### Structure of ACPB-bound hGGT1

**Figure 6:**
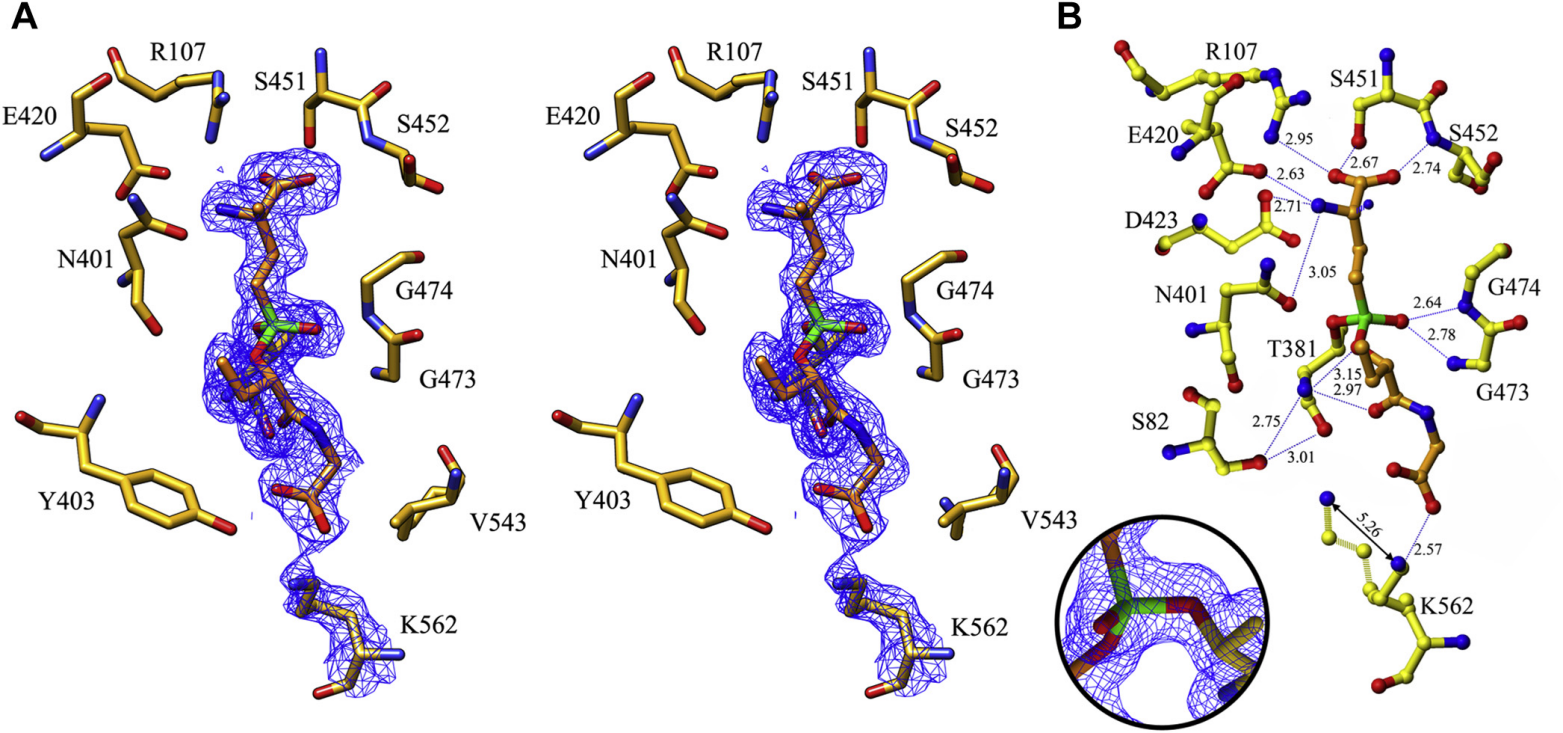
**ACPB in the active site of hGGT1 (6XPB).***A*, relaxed stereo presentation of the part of the final 2*F*_*o*_*-F*_*c*_ electron density map for the ACPB-bound hGGT1 structure that corresponds to the cleaved ACPB molecule and Lys-562. *Circle Insert,* the image in Figure 6*A* is rotated vertically approximately 90° clockwise and cropped to highlight the Thr-381-ACPB bond. The complete ACPB-Thr-381 image is shown in the supplementary information (Fig. S1). *B*, interactions of ACPB with hGGT1. Enzyme carbon atoms are colored *yellow*; ACPB carbon atoms are colored *orange*, oxygens are *red*, nitrogens are *blue*, and phosphate is *green*. Resolution is 1.74 Å.

**Figure 7:**
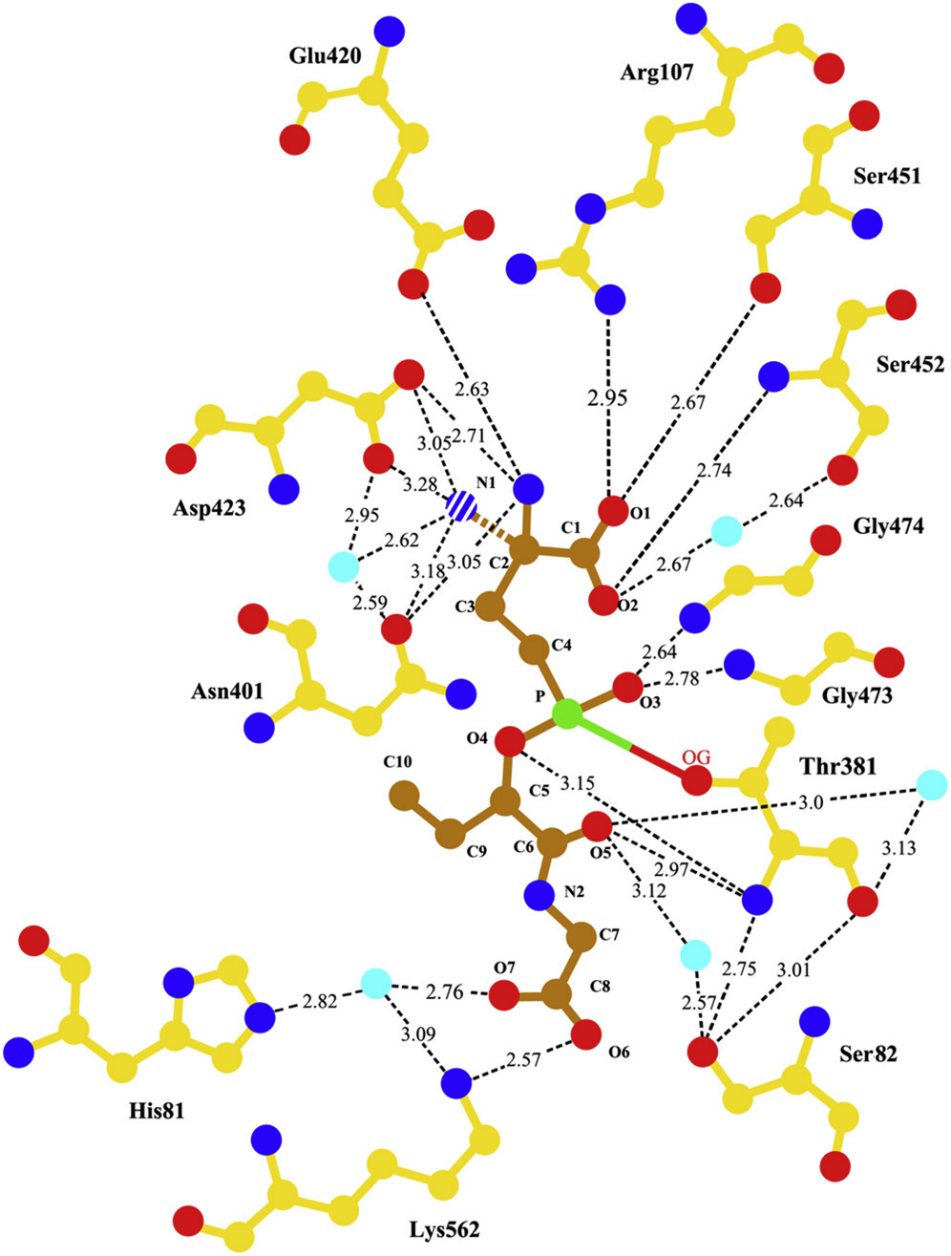
**LIGPLOT diagram of the interactions between hGGT1 and ACPB.** Enzyme carbon atoms are colored *yellow*; ACPB carbon atoms are colored *orange*, oxygens are *red*, nitrogens are *blue*, phosphate is *green*, and water molecules are *cyan*. Solid blue N1 is *Lα*-ACPB isomer. Striped blue N1 is *Dα*-ACPB isomer.

The GSH analog ACPB inhibits hGGT1 activity by forming a covalent bond with the enzyme, thereby inactivating it. hGGT1 was incubated overnight with a 60-fold molar excess of ACPB prior to crystallization, although even after just 1 h of incubation, no hGGT1 activity was detected in the mixture. Crystals were obtained and data were collected. Initially ten cycles of rigid body refinement were executed with the coordinates of apo-hGGT1 (4Z9O), (all water molecules and metal ions were removed), followed by ten cycles of restrained refinement. The structure of the ACPB-bound hGGT1 complex (6XPB) was solved at 1.74 Å resolution (Fig. 6, Fig. 7 and Fig. S1).

The difference Fourier maps showed residual electron density in the active site of the enzyme that extended from the binding site of the main-chain atoms of the Glu moiety of glutamate analog inhibitors toward Thr-381, the catalytic nucleophile, and further into a pocket below Thr-381. In the initial difference Fourier maps, the electron density for the region of ACPB that mimicked cysteinylglycine was about 30% weaker than for the glutamate portion of ACPB. This ratio was also true for the final electron density. This indicated that the cysteinylglycine region of the enzyme-bound ACPB was more flexible than the glutamate region. However, modeling the ACPB into the difference electron density was unambiguous. The structure of the ACPB-bound hGGT1 crystals showed that ACPB was covalently bound to the enzyme, and the phenoxy group on the phosphate of ACPB was no longer present (Fig. 3, Fig. 6, Fig. 7 and Fig. S1). We previously reported the structure (4ZBK) of hGGT1 crystalized with another phosphorous-based, glutamate analog that inhibits hGGT1, GGsTop (Fig. S2) (4). This structure showed that the leaving group of the inhibitor molecule was also the phenoxy group (oxybenzeneacetic acid), while its methoxy group remained bound to the phosphate (21). Based on the ACPB-bound hGGT1 structure, the phenoxy leaving group of ACPB was oriented in the active site in the same position as the retained methoxy group of GGsTop. These data indicate that the leaving group from these two phosphate-based inhibitors was not determined by their position in the active site of the enzyme. Rather, as suggested by Han and colleagues, the leaving group was the group with the weakest bond to the phosphate (17). For ACPB and GGsTop, the weakest is the PhO–P bond.

The ACPB used for inhibition of the enzyme was a mixture of diastereoisomers. There are two chiral centers in the molecule. The α-carbon atom of the glutamate moiety gives rise to *Lα*-ACPB and *Dα*-ACPB. The second chiral center, the phosphorus atom, gives rise to *Rp*-ACPB and *Sp*-ACPB. To identify which of the two isomers related to the phosphoryl center bound to the enzyme, we built models of all four diastereoisomers of ACPB (*LαRp*-ACPB, *DαRp*-ACPB, *LαSp*-ACPB, and *DαSp*-ACPB) using Ligand builder of COOT and fitted them into the active site of the enzyme. Nucleophilic attack and cleavage of the phenoxy group require that the phosphoryl group of ACPB be aligned in the active site of the enzyme in a way that allows close contact between the Thr-381 side-chain OG and the phosphorus atom of ACPB. Modeling showed that of the four isomers, both isomers of the α-carbon *Lα*-ACPB and *Dα*-ACPB could be fitted into the difference electron density. With the two other isomers, such an alignment is impossible for the *Rp* stereo-isomer due to clashes between the oxyanion-forming loop and the cysteinylglycine mimicking region of the inhibitor. Therefore, we concluded that only the *Sp* stereoisomers of ACPB bind the enzyme. This conclusion is in agreement with results of kinetic and modeling studies by Watanabe and colleagues (27). These investigators synthesized the four stereoisomers of the phosphate-based inhibitor GGsTop (Fig. S2). The chiral centers in GGsTop are the same as those in ACPB. They found that the *LαRp* and the *DαRp* isomers had no inhibitory activity. Based on the kinetic data and molecular modeling, these investigators concluded that only the *Sp* isomers of GGsTop were able to inactivate hGGT1. Refinement of our data showed that both *LαSp*-ACPB and *DαSp*-ACPB were bound in the hGGT1 crystals.

Initially, the Lα isoform was modeled into the difference electron density. With refinement of the model, it became clear that the structure contained a mixture of *Lα* and *Dα* forms of the product of the reaction. The *F*_*o*_*-F*_*c*_ difference Fourier map showed a clear positive density in a location that corresponded to the α-nitrogen of the glutamyl moiety in the Dα isoform of ACPB. In the final model with R_work_ and R_free_ of 15.3 and 18.9, respectively, the inhibitor was modeled as 70% in the *Lα* isoform and 30% in the *Dα* isoform. The only difference in the binding of the two isoforms was the interactions between the α-nitrogen of the glutamyl moiety and the enzyme (Fig. 6 and Fig. 7). In the *Lα* isoform, the glutamyl moiety of the inhibitor maintains the same interactions with the protein atoms as in all of our previously described hGGT1-substrate/inhibitor structures (Protein Data Bank 4GDX, 4Z9O, 4ZBK, 4ZC6, 4ZCG, 5V4Q). In the *Dα* isoform, the α-nitrogen (N1 shown as a stripped ball in Fig. 7) formed hydrogen bonds with OD1 atom of Asn-401 (3.18 Å), OD1 and OD2 atoms of Asp-423 (3.28 Å and 3.05 Å, correspondingly), and one water molecule (2.62 Å) that intermediates bonds with oxygens of both Asn-401 and Asp-423 (OD1). Watanabe and colleagues reported that the *LαSp* isomer of GGsTop had a k_on_ rate that was eight times higher than that of the *DαSp* isomer (27). Our structural data suggest that the difference in potency between the *LαSp* and *DαSp* isomers of these phosphate-based hGGT1 inhibitors is due exclusively to a difference in the affinity of the enzyme for the region surrounding the α-nitrogen of the glutamate group as the inhibitor begins to bind in the active site. There are no differences in the binding of the two isoforms of ACPB beyond the N1.

ACPB inhibits hGGT1 by forming a covalent bond within the active site of the enzyme. The data show that the phosphorous atom of ACPB formed a covalent bond with side-chain oxygen of Thr-381, the catalytic nucleophile for hGGT1-catalyzed reactions. This covalent bond with phosphorous is analogous to the covalent acyl bond that forms transiently between the C5 carbon of γ-glutamyl substrates and side-chain oxygen of Thr-381 during cleavage of the γ-glutamyl bond. In the ACPB-bound hGGT1 structure, one of the oxygens of the PO_3_ group is gone, displaced by the side-chain oxygen of Thr-381. The second oxygen atom of the PO_3_ group (O3 with a double bond to P) interacts via hydrogen bonds with the main-chain nitrogen atoms of Gly-473 and Gly-474 (2.78 Å and 2.64 Å, respectively). These two residues are part of the oxyanion hole. In ACPB-bound hGGT1, the oxyanion loop (Gly-473–Thr-475) is in one of the two conformations seen in our glutamate-bound hGGT1 structure (4GDX) reported previously (18). The loop is in the more open conformation. The binding of ACPB locks the active site into a conformation that corresponds to the hGGT1 enzyme–substrate tetrahedral intermediate.

Analysis of the ACPB-bound hGGT1 shows that within the region of ACPB that mimics cysteinylglycine, only three atoms (O5, O6, O7) interact with the enzyme (Fig. 7). Yet, this is the location of the most dramatic movement within the enzyme as substrates/inhibitors bind in the active site. O5, the carbonyl oxygen of the cys-like residue of ACPB, interacts with the *N*-terminal nitrogen of the nucleophile Thr-381 (2.97 Å). Through a water molecule, this atom also interacts with the side-chain OG atom of Ser-82 in one of its two conformations. Ser-82 had two conformations in this structure, but only one in the GSH-bound hGGT1. Through a second water molecule (3.0 Å), it also interacts with main-chain oxygen of Thr-381. O6, one of two oxygen atoms of the C-terminal carboxyl group of ACPB, formed a salt bridge with the NZ atom of the Lys-562 side chain (2.57 Å, Fig. 7). A comparison of the position of Lys-562 side chain in the apo-hGGT vs ACPB-bound hGGT1 shows the side chain repositioned inward toward the carboxy end of the inhibitor (Fig. 6). Indeed, the *F*_*o*_*-F*_*c*_ difference map showed a negative density at the site of the side chain of Lys-562 in the structure of apo-hGGT1 (used as a model) and positive density in the ACPB-bound hGGT1 structure at this new position. To model this movement, the chi2 and chi3 angles of the residue were changed from 167° and 177° to 57° and 64°, respectively. The result of the movement was displacement of the NZ atom by more than 5 Å. The second oxygen (O7) of the C-terminal carboxyl group of the inhibitor did not make any direct interaction with protein atoms. Its two interactions were via a single water molecule that intermediated interactions with the NE2 atom of His-81 and the NZ atom of Lys-562. The O–O distance between the water molecule and the carboxyl group of ACPB was 2.76 Å. The water molecule O-NE2 distance was 2.82 Å. The O–NZ distance was 3.09 Å.

The ethyl group in ACPB, which mimics the side chain of a cysteine residue (Fig. 3), was oriented toward the solvent and was not involved in any direct interactions with protein. The structure indicates that for GSH *S*-conjugates as substrates, the *S*-conjugate would be oriented toward the open cleft in the active site. There would not be any steric hindrance restricting the size of the conjugate group. This orientation of the substrate is consistent with the data from kinetic studies of hGGT1, which show similar binding and kinetics for GSH and for GSSG, which has two GSH molecules bound via the sulfur atoms (1). Further, hGGT1 cleaves a large number of GSH *S*-conjugates, including those with large bulky *S*-conjugates such as LTC_4_ (1).

When compared with the apo-hGGT1 structure, the structure of ACPB-bound hGGT1 has a larger active site cavity, shifts in the polypeptide backbone of the enzyme, and a major shift in the side chain of Lys-562. The superposition of 316 CA atoms of the large subunits of both structures (residues 36–375 with exclusion of residues with multiple conformations) gave *rms* deviation of 0.40 Å, with largest movement of 1.35 Å for residue Thr-183. When all of the CA atoms of the small subunit with a single conformation were superimposed, the *rms* deviation between the two structures was 0.40 Å with the biggest displacement of 1.61 Å for Thr-475 of the oxyanion hole forming loop. This loop had moved away from the bound ACPB molecule. As a result, the active site cavity was more open in the ACPB-bound hGGT1 structure than in the apo-hGGT1 structure. Also contributing to the opening of the active site cavity was the movement of an irregular part of the polypeptide chain (residues 401–421) across the active site from the oxyanion hole. The largest shift of 0.98 Å was registered for the CA atom of Tyr-403. Displacement of the loop bearing residues 79–82 of large subunit located behind the above-mentioned irregular region was also detectable. Aromatic side chains of His-81 and Tyr-403 that are part of these loops did not change their conformation upon inhibitor binding but moved as a rigid body together with the corresponding chains. The helices α7-α9 located above and to the right of the active site pocket (Fig. 1) were also shifted away from the active site. The expansion of the active site due to ACPB binding is demonstrated by the increase from 7.79 Å to 9.83 Å in the distance between the CA atoms of residues Gly-474 and Leu-402 in the ACPB-bound hGGT1 structure compared with the apo form. Similarly the distance between CA atoms Gly-473 and Tyr-403 increased from 9.59 Å to 11.67 Å.

### Realignment of the substrate and movement within the active site of hGGT1 as the acyl bond forms

A comparison of the structures of the GSH- and ACPB-bound forms of hGGT1 suggests that soaking the apo-hGGT1 crystals in GSSG resulted in the entry of GSH into the active site; however, the rigidity of the crystal prevented full accommodation and binding of the substrate in the active site. ACPB was bound to hGGT1 prior to crystallization and therefore revealed the structure of a fully bound GSH analog in the active site. It is not possible to cocrystallize GSH and hGGT1 because the enzyme rapidly cleaves all of the GSH. LSQ superposition of all 530 CA of atoms (35–375 and 381–569) of the GSH- and ACPB-bound hGGT1 structures gave *rms* deviation of 0.35 Å. If subunits are superimposed separately, then *rms* deviation is 0.37 Å for the large subunit and 0.24 Å for the small subunit. Comparison of the GSH- and ACPB-bound hGGT1 structures showed the largest shift in the CA atoms (excluding the N and C terminal few residues of the large subunit) for residues 170–208 that form helices α6, α7, α8, and β strands β5, β6, and β7. The largest shift was 1.18 Å between the CA atoms of the Asn-180 residues in the two structures. This shift was in part responsible for the opening of the active site cleft in the substrate-bound hGGT1 structure. The loops carrying His81 (residues 78–83) and Tyr-403 (401–406) also show noticeable differences, with largest shift of 0.65 Å for the CA atoms of His-81 residues. These elements of the structure form the left wall of the active site pocket (Fig. 1). As the substrate binds, these elements are shifted away from active site, thereby enlarging the space where the glycine group of the substrate binds.

We sought to further investigate the movement within active site as substrates bind and form a tetrahedral substrate–enzyme intermediate required for cleavage of the γ-glutamyl bond of the substrate. To visualize this dynamic process, we modeled GSH in the ACPB-hGGT1 structure and transitioned from the GSH-bound hGGT1 structure to the ACPB-bound hGGT1 structure containing the modeled GSH (Movie S3). Cross-orientation of GSH and ACPB molecules when all the CA atoms of the two complexes are superimposed is shown in Figure S3. The two ends of the compounds are located very close to each other. The main-chain atoms of both compounds occupied almost same position with the same network of hydrogen bonds to enzyme atoms. The spatial positions of glycine moieties in both structures are close; however, the orientation planes of the carboxyl groups in the two structures are almost perpendicular. The dynamic simulation shows concurrent movement of the NZ atom of Lys-562 and the formation of a second bond between the substrate and the oxyanion hole (new bond with Gly-474) (Movie S3). The simulation does not identify the driving force for the reorientation of the substrate as it becomes more fully anchored within the active site. One contributing factor could be the side chain of Lys-562. Its NZ atom forms a salt bridge with one of the carboxy oxygens of glycine as the NZ atom moves 5 Å.

In the GSH-bound hGGT1 structure, Thr-381 has two conformations. The distance between the CA atoms in the two conformations was 0.63 Å. The simulation shows that with Thr-381 in the A conformation, the reorientation of the substrate aligns the OG1 of Thr-381 and the C5 atom of GSH in close proximity. When the distance between OG of Thr-381 and C5 atom of the substrate is favorable for nucleophile attack on the C5 atom, a bond is formed, converting the enzyme–substrate complex into a tetrahedral intermediate. Not shown in this simulation is the resolution of the intermediate during which the γ-glutamyl bond of the substrate is hydrolyzed followed by the cleavage of the substrate–enzyme acyl bond. The rate-limiting step of the GGT1 reaction is the hydrolysis of the acyl bond (hydrolysis reaction), a process that is accelerated by the presence of a high concentration of acceptor molecules, such as glycylglycine, in which the γ-glutamyl group is transferred to the acceptor (transpeptidation reaction) (Fig. 2).

### Structural data and enzyme kinetics

Our structural data show the formation of hydrogen bonds and salt bridges between the substrate and the enzyme as the substrate enters the active site. As the substrate analog (ACPB) moves further into the active site and becomes covalently bound, the bonds between the γ-glutamyl portion of the substrate/inhibitor and the enzyme do not change. These bonds anchor the substrate in the active site. In contrast, the hydrogen bonds and salt bridges between the enzyme and the cysteinylglycine region of the substrate change, indicating their involvement in drawing the substrate deeper into the active site and orienting it for cleavage. When these structural data are analyzed in light of previously published kinetic data with hGGT1 mutants, the contribution of these bonds to the catalytic activity of the enzyme becomes apparent.

Enzymatic activity of hGGT1 is lost when Ser-451, Ser-452, Arg-107, or Asp-423 is mutated (24, 25, 26). Each of these residues interacts with the α-carboxyl or α-amino of the γ-glutamyl portion of the substrate. In our structures three atoms in the cysteinylglycine region of both GSH and ACPB formed hydrogen bonds or salt bridges with the enzyme. Those atoms are the main-chain oxygen of the cysteine (O4 of GSH, Fig. 5) and the two carboxy oxygens of glycine (O5 and O6 of GSH, Fig. 5). Based on molecular modeling, several groups of investigators suggested that Lys-562 is important in substrate binding (23, 28).

Our structures show that the side-chain NZ atom of Lys-562 interacts with one of the two carboxyl oxygens of glycine in the GSH-bound structure via a water bridge (intermediate in the process of substrate binding). In the ACPB-bound structure this nitrogen has moved 5 Å, forming a salt bridge with one of the oxygens and is bound via a water to the other (Figure 4, Figure 5, Figure 6, Figure 7). Two groups of investigators have mutated Lys-562 in hGGT1 and assayed the enzymatic activity of the mutants (23, 28). Hu and colleagues reported greater than 70% reduction in hydrolysis activity in K562N and K562Q (23, 28). Kamiyama and colleagues expressed a K562S mutant of hGGT1 and did not observe any reduction in hydrolysis activity (23, 28). These seemingly contradictory results can be explained based on the different substrates used by the two groups to assay hydrolysis activity.

**Figure 8:**
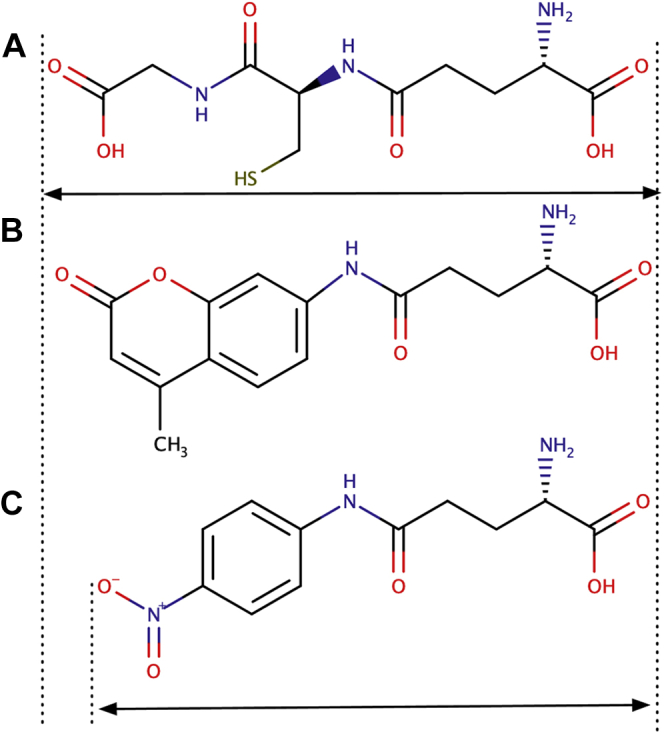
**Diagrams and relative lengths of hGGT1 substrates.***A*, GSH. *B*, γ-Glu AMC. *C*, L-GpNA.

Hu and colleagues used L-γ-glutamyl 7-amido-4-methylcoumarin (γGlu AMC) as a substrate (Fig. 8) (28). This compound has a carbonyl oxygen equidistant from the γ-glutamyl bond as the carboxy oxygen on the glycine moiety of GSH (Fig. 8). In contrast, Kamiyama and colleagues used γ-glutamyl *p*-nitroanilide (L-GpNA) as a substrate (Fig. 8) (23). This compound has nitro-oxygens that are a shorter distance from the γ-glutamyl bond than are the carboxy oxygens in GSH. Therefore, the carboxy oxygens of GSH and L-Glu AMC reach further toward the NZ of Lys-562 in the cysteinylglycine pocket than do the nitro-oxygens of L-GpNA. This suggests that the nitro-oxygens of L-GpNA could have much weaker interactions with the side-chain nitrogen of Lys-562. The weaker interaction would explain why mutation of Lys-562 did not affect the hydrolysis activity of the enzyme with L-GpNA. It would also predict a lower affinity of L-GpNA for the enzyme compared with GSH and GSH analogs. The kinetics of the hGGT1 hydrolysis reaction support this theory.

For the hydrolysis reaction catalyzed by hGGT1, the K_m_ of ɣ-Glu-AMC is 12.6 μM and the K_m_ of GSH is 10.6 μM (1, 17). Both of these substrates have oxygens that are located topologically such that they would reach far enough into the binding pocket to interact with the side-chain nitrogen of Lys-562. In contrast, for the hydrolysis reaction, the K_m_ of L-GpNA is 0.83 mM (12). Not only is the K_m_ of GSH 78-fold lower than of L-GpNA, but the V_max_ is 1000-fold higher, 6.3 μM/min/nM vs 6.5 mM/min/nM, respectively (1, 12, 23). In addition, investigators developing inhibitors of hGGT1 based on sulfur or phosphate derivatives of γ-glutamyl compounds have observed that adding a carboxy group at a position equivalent to the C-terminal carboxy of GSH increases the inhibitory activity of the compound more than 100-fold (17, 29). Detailed kinetics showed that effect of the addition of this group was primarily due to increased affinity of the inhibition for the active site of the enzyme (17).

There are also kinetic data with a Y403A mutant of hGGT1 (28). Our GSH-bound hGGT1 structure shows a hydrogen bond between the phenolic oxygen of Tyr-403 and one of the carboxyl oxygens on the glycine of GSH. The Y403A mutant of hGGT1 hydrolyzed γ-Glu-AMC at only 50% the rate of the wild-type enzyme. We did not observe hydrogen bonds between ACPB and Y403 of hGGT1, suggesting that the role of this residue may be in initially tethering the substrate to the active site. These authors did not evaluate the activity of the Y403A mutant in the hydrolysis of L-GpNA; however, studies with hGGT5 indicate that the Y403 may be essential for cleaving L-GpNA.

GGT5 is a member of the GGT1 family (30). Human GGT5 has 41% identity with hGGT1. Like GGT1, it is an Ntn hydrolase that expresses as a single polypeptide chain and autocleaves into two subunits. The GGT1 residues that bind the γ-glutamyl portion of the substrate (Ser-451, Ser-452, Arg-107, Glu-420, Asp-423, and Asn-401) are all conserved in hGGT5. Also conserved are the residues of the oxyanion hole (Gly-473 and Gly-474) and the catalytic nucleophile Thr-381. The K_m_ of GSH for hGGT1 and hGGT5 is the same (10.6 μM and 10.5 μM, respectively). hGGT5 hydrolyzes GSH and GSH-conjugates, although at only 3% of the V_max_ of hGGT1 (1). Differences between hGGT1 and hGGT5 are seen in the region of the active site where the cysteinylglycine portion of the substrate binds. In hGGT5, Lys-562 is conserved, but His-81 is a glutamine and Tyr-403 is a proline. hGGT5 cannot cleave L-GpNA (31). While there are many nonconserved amino acids between hGGT1 and hGGT5, the fact that both catalyze the hydrolysis of GSH but only GGT1 catalyzes the hydrolysis of L-GpNA suggests that Y403 and H81 play an important role in the affinity for the active site of hGGT1 of substrates like L-GpNA that cannot reach the Lys-562.

In the presence of high concentrations of acceptor amino acids and dipeptides, GGT1 catalyzes a transpeptidation reaction (Fig. 2). While the concentration of acceptors is rarely sufficient *in vivo* to stimulate transpeptidation activity of hGGT1, analysis of the kinetics of the transpeptidation reaction can provide insight into the catalytic activity of hGGT1. Glycylglycine is the most commonly used acceptor in kinetic assays, with a K_m_ of 10 mM (12). With glycylglycine as the acceptor, the K562S mutant had only 2% of the transpeptidation activity of the wild-type hGGT1 (23). These studies were done with L-GpNA. With L-GpNA, this mutant has 100% of the hydrolysis activity of the wild-type enzyme, but only 2% of the transpeptidation activity, indicating a critical role for Lys-562 in the transpeptidation reaction. With ɣ-Glu AMC as a substrate, K562N and K562Q mutants of hGGT1 had less than 2% of the transpeptidation activity of the wild-type enzyme (28). Y403A mutant had less than 10% of the transpeptidation activity of the wild-type hGGT1 (28). Modeling glycylglycine into our GSH-bound hGGT1 structure revealed a binding mode for this acceptor in which, following release of the cysteinylglycine portion of the substrate, glycylglycine could be tethered to the active site by Lys-562 and Tyr-403, initiating the attack of its α-nitrogen on the C5 atom of the γ-glutamyl-enzyme intermediate. hGGT5 has Lys-562 and catalyzes a transpeptidation reaction (1). The structure of *Escherichia coli* GGT1, which lacks a Lys-562, provides further insight into the critical role of residues within the active site.

*E. coli* GGT1 has 33% identity with hGGT1. The structure of ACPB-bound *E. coli* GGT1 (5B5T) has been published (23). It shows the same network of interactions between the enzyme and the α-carboxy and α-nitrogen of the glutamate moiety of ACPB that we observed in ACPB-bound hGGT1 (23). The bonds with the two glycines of the oxyanion hole and the catalytic threonine are also the same for ACPB bound to *E. coli* and to hGGT1. However, no specific interactions between the enzyme and the cysteinylglycine portion of ACPB were observed in the *E. coli* GGT1 structure. Of the three residues that bound to ACPB in the cysteinylglycine region in our ACPB-bound hGGT1 structure (H-81, Ser-82, and Lys-562), none are conserved in *E. coli*. *E. coli* GGT1 catalyzes a transpeptidation reaction in which the K_m_ values for GSH and L-GpNA are both 35 μM and the K_m_ for glycylglycine as an acceptor substrate is 590 mM (32). These values compare with 10.6 μM, 830 μM, and 10 mM respectively for hGGT1. Surprisingly, ACPB inactivates *E. coli* GGT1 and hGGT1 at the same rate (k_on_ 80 vs 75 M^−1^ s^−1^, respectively) (17). There are many differences between the active sites of *E. coli* GGT1 and hGGT1 that could contribute to the differences in K_m_ values for substrates yet the same k_on_ rate for ACPB. Overall, the cysteinylglycine binding pocket of *E. coli* GGT1 has a broader specificity for substrates and inhibitors than it has in hGGT1 (17).

### Summary

Our structural data enhance the understanding of the interactions of substrates and inhibitors with the active site of hGGT1. Upon initial binding of GSH, both Asn401 and Thr-381 form two hydrogen bonds, each with atoms of GSH that span the γ-glutamyl bond. The data confirm the role of Tyr-403 and Lys-562 in substrate binding and identify His-81 and Ser-82 as interacting with the substrate. The structures reveal that three different atoms of hGGT1 interact with the carboxy oxygen of the cysteine of GSH. In-depth comparison of the apo-GGT1 structure with GSH-bound hGGT1 and ACPB-bound hGGT1 shows an enlargement of the active site as the substrate initially binds. The loop containing the residues of the oxyanion hole is displaced. Multiple changes are seen concurrent with the entry of the substrate deeper into the active site cleft. The side-chain nitrogen of Lys-562 that binds the substrate moves 5 Å. The substrate reorients and a new hydrogen bond is formed between the substrate and the oxyanion hole. Thr-381 is locked into a single conformation as an acyl bonds forms between the substrate and the enzyme. These interactions provide a molecular understanding of the kinetic changes observed with hGGT1 mutants and will aid in the design of specific and potent inhibitors of hGGT1.

## Experimental procedures

### hGGT1 expression and purification

For crystallization studies, hGGT1 (P19440), the V272A variant, was expressed in *Pichia pastoris* strain X-33, purified and deglycosylated as described previously (18). hGGT1 autocleaves into two subunits: the large subunit (amino acids 1–380) and the small subunit (amino acids 381–569). The first 27 amino acids of the large subunit, the transmembrane region that anchors the protein into the cell membrane, were omitted from our construct.

### Synthesis of 2-amino-4-(((1-((carboxymethyl)amino)-1-oxobutan-2-yl)oxy)(phenoxy)phosphoryl)butanoic acid (ACPB)

ACPB was first synthesized by Han and colleagues and is compound 4 in the reference (17). For our experiments, ACPB was synthesized in the laboratory of Dr Anthony Burgett at the University of Oklahoma, Norman, Oklahoma, as previously described (17). The mixture of diastereomers was used for biological experimentation. ^1^H NMR (300 MHz, DEUTERIUM OXIDE): δ ppm 7.41–7.05 (m, 5H), 4.81 (m, 1 H), 4.00–3.77 (m, 3H), 2.22–2.10 (m, 3 H), 1.90–1.55 (m, 5 H), 0.87 (m, 6 H), 0.90–0.76 (m, 3H). HRMS (+ESI): calculated for C_16_H_23_N_2_O_8_P[M + H^+^]: = 403.1265; found 403.1268 Δ = 0.74 ppm. See supporting data for NMR and mass spectrometry spectra (Fig. S4, Fig. S5).

### Crystallization

For studies with GSH, crystals of apo-hGGT1 were grown at room temperature by vapor diffusion with the hanging drop method as described previously (21). The protein concentration was 2.5 mg/ml, the reservoir contained from 20% to 25% (w/v) PEG3350, 0.1 M NH_4_Cl, and 0.1 M Na cacodylate at pH 6.0. The crystals were soaked in 25% (w/v) PEG3350, 0. 1 M NH_4_Cl, and 0.1 M Na cacodylate at pH 6.0 supplemented with 200 mM GSSG for 2 h at room temperature prior to cryoprotection.

The ACPB-bound complex was prepared by incubating hGGT1 with ACPB at 4 °C. One microliter of 0.3 M ACPB (in a 35/65 methanol/water mixture) was added to 49 μl of a 5 mg/ml solution of hGGT1 in 50 mM HEPES pH 8.0, 0.5 mM EDTA, and 0.02% (w/v) NaN_3_. An aliquot taken after 1 hour of incubation had no hGGT1 activity as measured by the GGT1 transpeptidation assay (see below). The mixture was then incubated overnight at 4 °C. The following day, hanging drops were made by mixing 1.0 μl of the ACPB-hGGT1 mixture, 1.0 μl water, and 1.3 μl of reservoir solution containing 20–25% (w/v) PEG3350, 0.1 M Na cacodylate at pH 6.0, and 0.1 M NH_4_Cl. Drops were immediately seeded with micro-crystals of apo-hGGT1 (21). After 2 days the drops were seeded again. After a week of growth, 20 μl of glycerol was added to the reservoir solution to promote further growth of crystals. Six days after the addition of the glycerol, the crystals were harvested.

For cryoprotection, the crystals of both complexes were quickly passed through the reservoir solution supplemented to a final concentration of 15% (w/v) PEG1500 with 200 mM GSSG or 6 mM ACPB, correspondingly. Crystals were vitrified by dipping them into liquid nitrogen.

### Data collection and processing

Diffraction data for the GSH-bound hGGT1 crystals were collected remotely at beamline BL12-2 at the Stanford Synchrotron Radiation Light Source (SSRL, Menlo Park, CA) using a Pilatus 6M detector, with an X-ray wavelength of 0.9792 Å. The data were processed by XDS and scaled with Aimless from the CCP4 program suite (33). Diffraction data for the ACPB-bound hGGT1 crystals were collected at 100K at the X25 beamline of the National Synchrotron Light Source (BNL, Upton, New York). The data were collected at 1.1 Å on a Pilautus 6M detector, and the resulting diffraction patterns were processed using the HKL2000 suite (34).

### Structural determination, refinement, and analysis

The space group and unit cell parameters of the crystals (Table 1) were isomorphous to those from our published structures of hGGT1 in the apo form and in complex with glutamate and inhibitors that are glutamate analogs (Protein Data Bank 4GDX, 4Z9O, 4ZBK, 4ZC6, 4ZCG, 5V4Q). The structures were solved by difference Fourier maps. The coordinates were refined using Refmac (35). The structures were analyzed and corrected using COOT (36). Quality metrics of the final structures were evaluated using COOT and the validation server of the PDB. The statistics are shown in Table 1.

### Animation

The GGT binding and transition animation was created using Chimera, developed by the Resource for Biocomputing, Visualization, and Informatics at the University of California, San Francisco (37). The molecular surfaces produced and rendered by Chimera were created with embedded software from the MSMS package (38). The movies were constructed from three coordinate files (4Z9O, 6XPC, and 6XPB). To simplify the view and relevant dynamics, only the coordinates of the protein atoms, GSH, ACPB, and five relevant water molecules were selected to create the movies (Fig. 5 and Fig. 7). Only the most relevant conformation was kept for residues with alternate conformations. The five water molecule coordinates from 4Z9O were selected according to the position of water molecules in 6XPC. All water molecules were then assigned an arbitrary chain label, matched residue indices, and linked to a nearby residue of the corresponding protein by a hidden pseudobond. The GSH molecule in tetrahedral complex with the enzyme was modeled based on coordinates of ACPB in bound state with GGT by atom-to-atom matching of the GSH molecules with the ACPB molecules. GSH and ACPB-modeled GSH were then also linked to Asn-401 by a hidden pseudobond. Morph conformations were then generated from 4Z9O to 6XPC and from 6XPC to 6XPB. Distances between pairs of relevant atoms were monitored by built-in distance functions.

All procedures were done from command line prompt or script.

## Data availability

All of the structural data are available in the RCSB Protein Data Bank under the accession numbers: 6XPC and 6XPB. All other data are contained within the article and Supporting Information.

## Conflict of interest

The authors declare that they have no conflicts of interest with the contents of this article.
