# Supplementary material for: Crystal structures of glutathione- and inhibitor-bound human GGT1: critical interactions within the cysteinylglycine binding site
Source: J Biol Chem. 2020 Nov 22;296:100066. doi: 10.1074/jbc.RA120.016265 (PMC7949050; doi:10.1074/jbc.RA120.016265)
Supplement: Table S1 and Figures S1–S5 [file mmc4.pdf]

Crystal structures of glutathione- and inhibitor-bound human GGT1: Critical interactions within the cysteinylglycine binding site

Simon S. Terzyan, Luong T. Nguyen, Anthony W.G. Burgett, Annie Heroux, Clyde A. Smith, Youngjae You, Marie H. Hanigan*

* Corresponding author: Marie Hanigan   Email: marie-hanigan@ouhsc.edu

This PDF file includes:

Table S1: Sequence of secondary structural elements in hGGT1
Figure S1: Electron density map for the ACPB-bound in the active site of hGGT1
Figure S2: Structure of GGsTop
Figure S3: Superposition of GSH and ACPB molecules from corresponding complex structures
Figure S4: 400 mHz ^{1}H-NMR of crude ACPB in D_2O
Figure S5: 400 mHz ^{1}H-NMR of crude ACPB in D_2O

Other supporting information for this manuscript includes the following:

Movie S1: Initial binding of GSH to apo-hGGT1
Movie S2: Movement of the oxyanion loop and formation of additional interactions between hGGT1 and GSH
Movie S3: Conformational changes in hGGT1 and GSH to form the tetrahedral enzyme-substrate complex

**Table S1**. Sequence of secondary structural elements in hGGT1					
Secondary Structural Element	Starting Residue #	Final Residue #	Secondary Structural Element	Starting Residue #	Final Residue #
β1	37	39	α1	48	60
β2	42	45	α2	64	78
β3	88	95	α3	122	126
β4	100	106	α4	137	148
β5	168	170	α5	153	166
β6	196	198	α6	172	180
β7	200	203	α7	182	186
β8	207	209	α8	188	195
β9	259	261	α9	211	223
β10	265	269	α10	226	230
β11	272	277	α11	234	243
β12	381	387	α12	250	255
β13	393	399	α13	283	295
β14	456	460	α14	306	327
β15	465	472	α15	339	345
β16	504	505	α16	348	355
β17	513	515	α17	477	489
β18	534	536	α18	495	500
β19	543	550	α19	521	529
β20	553	557			
β21	567	568			
η1	129	131	η4	420	423
η2	300	303	η5	439	441
η3	366	369			

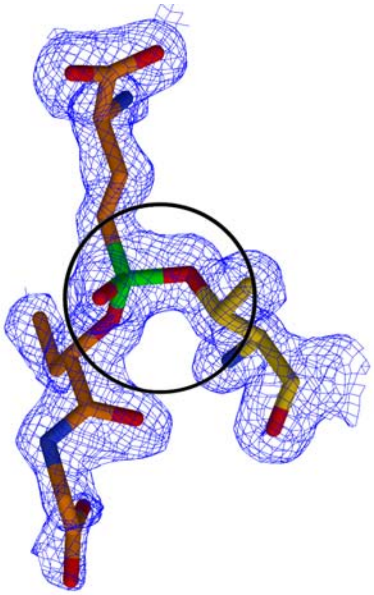

Fig. S1. Electron density map for the ACPB bound in the active site of hGGT1. The final $2F_o$-F_c electron density map for the ACPB bound in the active site of hGGT1 (6XPB) shown in Figure 6. The ACPB molecule has been rotated vertically approximately 90° clockwise relative to the image in Figure 6A. The circle highlights the Thr381-ACPB bond and is included as an insert in Figure 6. Enzyme carbon atoms are *colored yellow*; ACPB carbon atoms are *colored orange*, oxygens are *red*, nitrogens are *blue*, and phosphate is *green*. Resolution is 1.74 Å.

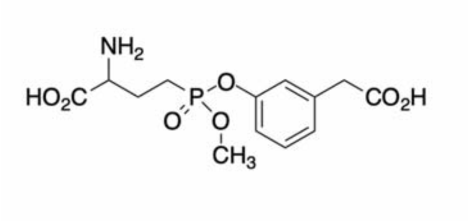

Fig. S2. Structure of GGsTop

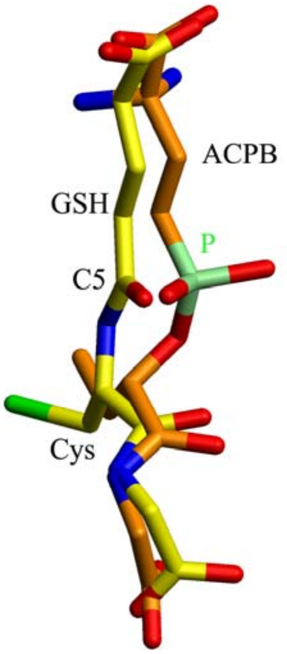

Fig. S3. Superposition of GSH and ACPB molecules from corresponding complex structures. The carbon atoms are colored *yellow* for GSH and *orange* for ACPB, oxygens are *red*, nitrogens are *blue*, the phosphorus atom is *pale green*, and the sulfur atom is *bright green*. The cysteine residue (Cys) and C5 atom of the GSH molecule are marked.

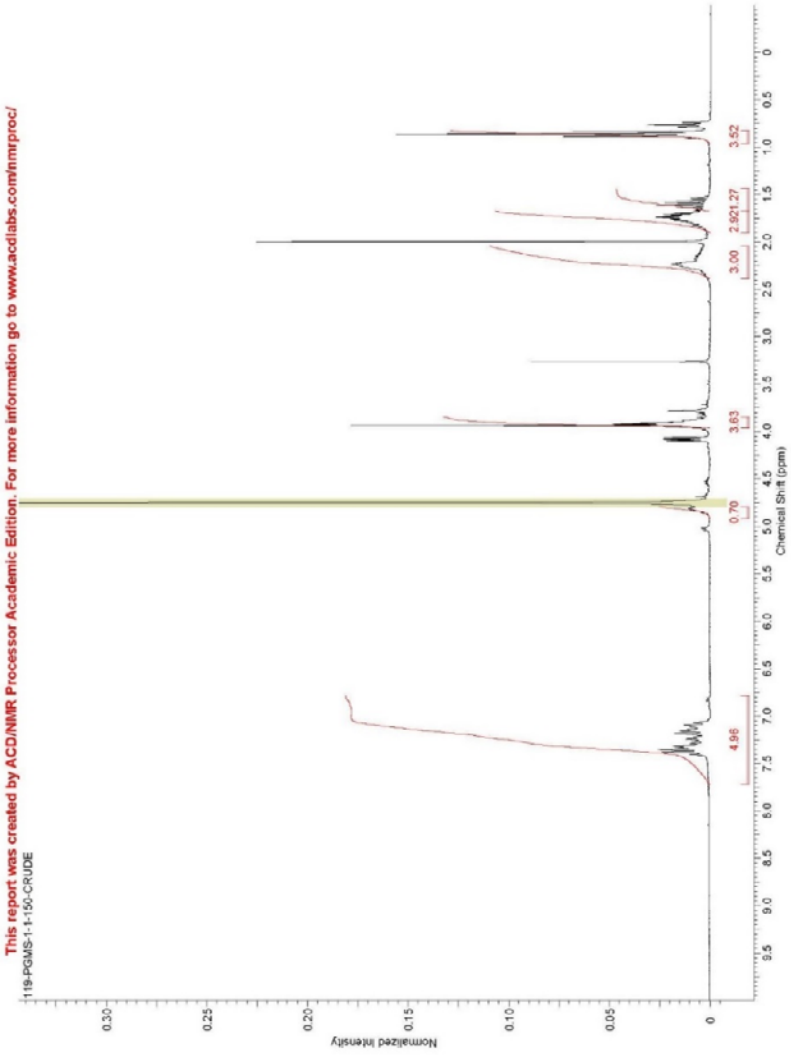

Fig. S4. 400 mHz ¹H-NMR of crude ACPB in D_2O.

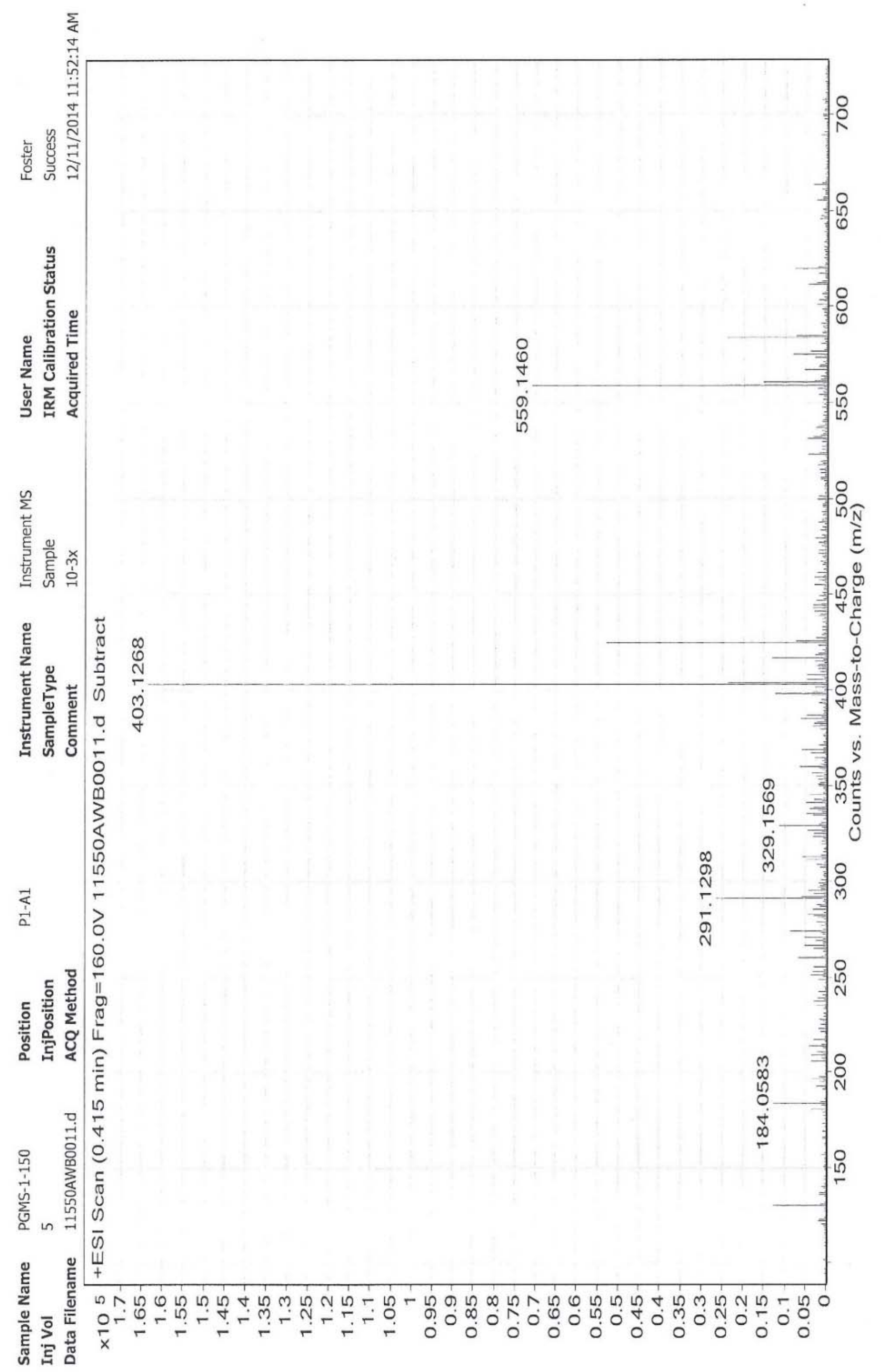

Fig. S5. 400 mHz ^{1}H-NMR of crude ACPB in D_2O.
